# A Case of Myelitis, Consequent upon Meningitis Cerebro-Spinalis, with Double Sensation, and Its Graphic Representation

**Published:** 1882-10

**Authors:** Otto Seiffert

**Affiliations:** First Medical Section of the Julius Hospital at Wurtzburg


### Original Translations.

A CASE OF MYELITIS, CONSEQUENT UPON MENINGITIS CEREBRO-SPINALIS, WITH DOUBLE SENSATION, AND ITS GRAPHICAL REPRESENTATION. By OTTO SEIFFERT, M.D., First Medical Section of the Julius Hospital at Wurtzburg. Translated by EDMUND A. BOSS.

The symptoms of double sensation as a complication in tabes have been mentioned in the writings of E. Remak (previously by Leyden), and Naunyn. The latter described in an article a peculiar disturbance of sensation, which differed materially from the experience of Remak.

While in Remak's recorded case the patient, on having his foot sole pricked, first experienced any sensation three seconds after the act was completed, in that of Naunyn (which was probably tabes dorsalis) a like peculiarity occurred, followed in about two seconds by a repeated sensation of increased and abnormal intensity. Naunyn has likewise stated that this phenomenon was observed by him in three other cases. In two of these cases it probably occurred in conjunction with tabes dorsalis, and in the third with myelitis.

This phenomenon Naunyn observed especially in the feet; there was even once a second repetition of the original sensation; generally of two sensations the second was the more prolonged and painful; frequently, however, this order was reversed, the first being longer and more painful. As long as this double sensation continued distinct (*i. e.*, while the interval between the two could be appreciated), so long could the sensation be localized, and so long was the second referred to the same locality as the first. In all of Naunyn's cases, this power of repeating an original sensation lasted but a few days.

Naunyn, in describing these cases of double sensation, inclined toward the theory of Charcot, called Charcot's Dysæsthesia, *i. e.*, that the patient feels at the place of contact a sharp pricking, which, as it is transmitted inward, becomes more obtuse, and finally, at the terminus, is sharp again, as at the beginning of the sensation.

Fischer has also often observed this phenomenon of double sensation mentioned by Remak, and is convinced that it is not infrequent, but differs in his experience from the observation of Naunyn. In a later article Fischer records, among others, a case of double sensation, viz:

A patient thirty-eight years old, afflicted with tabes, came under my care. The second sensations were the reverse of those recorded by Remak, being less painful and shorter in duration, and moreover, the first sensation being experienced two and a half seconds after impregnation, followed by a short period of quiescence, which was in turn succeeded by a feeling ill-defined, as though the foot had simply been touched. Remarkable to say, the intervals between the two sensations decreased until not any interval was distinguishable according to the method employed by Fischer. Below I record a case that came under my notice, and which, according to the testimony of many, merits attention.

He was a man æt. twenty-three, who up to that time had always been in good health, and had lived in comfortable circumstances. Patient became unwell February 23, 1880, while serving then in the army. He returned on the aforesaid day from field manœuvres to the barracks with headache, especially severe in the region of the brow and anterior superior portion of the head; at the same time he complained of feeling very cold. During the night he had a severe attack of chills; headache increased; patient became dizzy; next day he became delirious, and remained in this state till the second week of his sickness. In the meantime his lower extremites began to contract, and patient was obliged to lie in bed with his knees drawn up, voluntary movements being impossible. At the same time his urine was retained, and catheterization became necessary. Three weeks later a serious catarrh of the bladder set in; this was treated with injections of salicylic acid. From this time patient began to suffer from incontinence of urine.

After some time he became able to flex his knees, and in the middle of May patient first attempted to walk. His condition improved, and he was able, with the assistance of a nurse, to walk around a room.

In the middle of June patient had a relapse, and was again constantly in bed, and severe muscle cramps caused him great pain. At first only the muscles of the hips were affected in this way; patient felt as though the muscles of his hips were drawn together, his chest tightly laced, and as if his body were elevated, so that on moving he fell several times out of bed. These cramps lasted from a quarter to half an hour, and occurred several times a day. About the middle of August they decreased in virulence, and cramps of similar character occurred in his legs, chiefly in the night. His legs were drawn up toward his abdomen, and were only prevented from remaining in this position by the continued efforts of a nurse. On account of these cramps, the rest of the patient at night was much disturbed; they being separated by an interval of not more than half an hour.

From the middle of September these intervals became longer; first the cramps every hour, then only once a day, so that in a few months they returned only after a number of days; then they would be absent for a number of days again. During the day they would hardly be noticeable, and patient, if awake, could overcome them, and if they occurred at night, he would summon the nurse, who easily straightened his limbs.

Since the commencement of these cramps, indications of impending paralysis—difficulty in moving the upper and lower extremities, and inability to help himself—have become evident, so that patient is entirely unable to leave his bed.

Since December, 1880, constant constipation, which only powerful hydrastic cathartics could relieve; since that time patient has lost all desire to evacuate his bowels.

Since October, passage of calculi, varying in size, with urine; most of them the size of cherry-pits; two of them the size of a bean. In the months last past all these symptoms are constant; evidence of paralysis in the extremities, bladder, rectum; and cramps in the legs nightly.

Patient was received at the Julius Hospital, November 6, 1881. Status: patient tall; appearance pale; anæmic; no change in the inter-thoracic organs; muscles of hands and forearms atrophied; grasp of the fingers synchronous, but that of the right side less firm than that of the left; strength in both hands very deficient. In the right elbow furrows that can be pretty well smoothed out, though patient complains on stretching out his arm of pain in the muscles of the arm. On bending arm, left side appears stronger; stretching of the arm impossible. Movements in the right shoulder-joint freer, those of the left indifferent. Reflex nervous action of the upper extremities considerably increased; left more than the right. External side of the right arm, on slight touching and slight pricking with needle, shows no sensation; the internal part shows normal feeling and sensation of pain. On the left arm there are no parts incapable of receiving and conducting impressions. The lower extremities are stiff and inflexible, and pressed against the side of the bed.

On the right side, only feeble action in hips and knee-joints; on the left increased activity of movement in the same.

Passive movements difficult, and muscular contractions can only be overcome with difficulty, and on using more force patient complains of severe pains in his legs.

Reflex nervous action of patella, tibialis anticus and tendo Achillis greatly increased, and increase is bilateral.

No abnormality of sensation discoverable in lower extremities. However, by testing in many places, we find the reflex action to be a little tardy.

The muscles about the hips have in part also lost their power of sensation, so that the patient can only sit up in bed with great difficulty; voluntary movements of the hips totally impossible.

Abdomen swollen, nowhere irritable. Pulse soft, frequent (120), regular. Urine constantly dripping, so that patient is obliged to constantly use a receiver. Urine cloudy, alkaline in reaction, and contains a small amount of albumen. Calculi, well rounded, and composed of ammonio-magnesia phosphate, are found in first part of the urine discharged, and are about pin's head in size. The urinary sediment contained an exceedingly large number of crystals of ammonio-magnesia phosphate.

November 8. Evacuation of fæces difficult. Urine large in amount, 2000-2200 c.c., and of continued alkaline reaction. During the first few days, a large quantity of calculi, some as large as a bean; most of them the size of a pin's head or a split pea, were discharged with the urine.

November 17. Urine for the last few days free from calculi. Incontinence and alkaline reaction continued. Quantity large, 2400-2700 c.c. Faradaic stimulation of the muscles of the upper extremities produces little contraction, and galvanic excitation shows likewise greatly decreased action of the muscles. Galvanic stimulation of the lower extremities demonstrates similarly their diminished power of contraction, and with an intermittent Faradaic current, only feeble, long-continued twitchings were obtained.

November 18. Patient was brought into clinic to-day. In addition to what has already been found on examination before, there are several atrophied spots on his hands; strongly marked furrows on his palms and near the origin of the nails. To-day, for the first time, a singular and remarkable phenomenon was observed in the lower extremities. On pricking the soles and external borders of his feet to ascertain his power of sensation, it was discovered that a single impregnation produced two reflex twitchings and two sensations of pain. The reflex action and the perception of the sensation occurred together. Upon pricking, there was produced a reflex action, and synchronously with it an exclamation of pain; after a short pause, a second reflex action, with a second exclamation of pain. On minutely interrogating the patient, it was ascertained that between the two reflex actions, with their attending outcries, there was no continuing pain.

The treatment up to this time consisted in galvanization of the spinal region, use of Wildunger water, good diet and warm baths. Another symptom is to be mentioned, namely, that cramps in his legs often recurred during the night. Patient used to wake up from a sound sleep in deep pain, which disappeared on changing the painfully cramped position of his legs. The nurses were obliged to stretch his limbs with considerable force in order to afford the patient relief. The following prescription was used for these cramps:

| | | |
|---|---|---|
| ℞. | Calc. brom | 8.0. |
| | Calc. iod | 2.0. |
| | Aqua distil | 150.0. |
| | Syr. Spl | 20. |
| D. S. | 3 tablespoonfuls daily. | |

November 30. Urine steadily of alkaline reaction, little albuminous; quantity of urine per diem varies from 26-3400 c.c. On account of constipation, cathartics requisite from time to time. Double sensation can still be obtained on the foot sole. When two impregnations with a needle are made rapidly in succession, three sensations of pain are experienced by the patient. If a short time elapse between the two impregnations, he experiences four sensations of pain. The cramps in his legs still continue.

December 15. Patient subjectively little improved. Paralysis of the bladder and sphincter unchanged. No calculi have been lately passed. The warm baths are well borne by the patient, and seem to greatly benefit him. In place of the prescription above mentioned, patient receives every evening a large dose of potass. bromide. Double sensation on foot sole still continues.

December 25. Double sensation still present. The cramps in the lower extremities occur more rarely, and are less painful in character. Patient is made daily to sit in a chair, and to perform simple gymnastic exercises.

January 2. Double sensation unchanged. First attempt was made to day to illustrate graphically this phenomenon; concerning its success and termination see below.

February 1. During the past month bladder and rectal complications show an apparent improvement. Urine continues alkaline, but contains only a small amount of sediment, of pus and ammonio-magnesia phosphate. Calculi are no longer discharged. Power of movement in the hands and fingers much improved; cramps in lower extremities continue; double sensation likewise continues.

February 6. Within the last few days patient had an attack of renal colic; high fever, pain in the region of the left kidney, which terminated after a discharge of urinary calculi. Patient will be taken away to-day by his relatives.—From the *Weiner Medizinsche Wochenschrift.* No. 26, 1882.

